# Circle drawing as evaluative movement task in stroke rehabilitation: an explorative study

**DOI:** 10.1186/1743-0003-8-15

**Published:** 2011-03-24

**Authors:** Thijs Krabben, Birgit I Molier, Annemieke Houwink, Johan S Rietman, Jaap H Buurke, Gerdienke B Prange

**Affiliations:** 1 Roessingh Research and Development, Roessinghsbleekweg 33B, Enschede, the Netherlands; 2 Department of Rehabilitation, Nijmegen Centre for Evidence Based Practice, Radboud University Nijmegen Medical Centre, Reinier Postlaan 4, Nijmegen, the Netherlands; 3 Faculty of Electrical Engineering, Mathematics and Informatics, University of Twente, Drienerlolaan 5, Enschede, the Netherlands; 4 Rehabilitation Centre 'het Roessingh', Roessinghsbleekweg 33, Enschede, the Netherlands

## Abstract

**Background:**

The majority of stroke survivors have to cope with deficits in arm function, which is often measured with subjective clinical scales. The objective of this study is to examine whether circle drawing metrics are suitable objective outcome measures for measuring upper extremity function of stroke survivors.

**Methods:**

Stroke survivors (n = 16) and healthy subjects (n = 20) drew circles, as big and as round as possible, above a table top. Joint angles and positions were measured. Circle area and roundness were calculated, and synergistic movement patterns were identified based on simultaneous changes of the elevation angle and elbow angle.

**Results:**

Stroke survivors had statistically significant lower values for circle area, roundness and joint excursions, compared to healthy subjects. Stroke survivors moved significantly more within synergistic movement patterns, compared to healthy subjects. Strong correlations between the proximal upper extremity part of the Fugl-Meyer scale and circle area, roundness, joint excursions and the use of synergistic movement patterns were found.

**Conclusions:**

The present study showed statistically significant differences in circle area, roundness and the use of synergistic movement patterns between healthy subjects and stroke survivors. These circle metrics are strongly correlated to stroke severity, as indicated by the proximal upper extremity part of the FM score.

In clinical practice, circle area and roundness can give useful objective information regarding arm function of stroke survivors. In a research setting, outcome measures addressing the occurrence of synergistic movement patterns can help to increase understanding of mechanisms involved in restoration of post stroke upper extremity function.

## Background

### Introduction

Stroke is described as "an extremely complex breakdown of many neural systems, leading to motor as well as perceptual, cognitive and behavioral problems" [1]. Motor problems of the upper extremity following stroke include muscle weakness, spasms, disturbed muscle timing and a reduced ability to selectively activate muscles. Many stroke survivors move in abnormal synergistic movement patterns that already have been described decades ago [2,3]. More recent studies of Beer [4-6] and Dewald [7-9] showed strong coupling of the shoulder and elbow joint in stroke survivors in both isometric and dynamic conditions.

Six months after stroke, motor problems are still present in the majority of stroke survivors [10], limiting their ability to perform activities of daily living (ADL). Post stroke rehabilitation training aims to regain (partly) lost functions by stimulation of restoration or promoting compensational strategies, in order to increase the level of independence. During rehabilitation training movements are practiced preferably with high intensity, in a task-oriented way, with an active contribution of the stroke survivor in a motivating environment where feedback on performance and error is provided [11].

### Robotics

A promising way to integrate these key elements of motor relearning into post stroke rehabilitation training is the use of robotic systems. Systematic reviews indicated a positive effect on arm function after robot-aided arm rehabilitation training [12,13]. Six months after training, the effect of robotic training is at least as large as the effect of conventional training [14].

Besides training, robotic rehabilitation systems can be valuable tools for evaluation purposes. Quantities of body functions concerning movement performance [15] can be measured objectively with integrated sensors of many robot systems. Objective measurement of motor performance in stroke survivors is important to study the effectiveness of different rehabilitation training programmes, in order to identify the most beneficial approaches. The use of objective outcome measures, strongly related to affected body functions and structures, can help to understand the mechanisms that are involved in restoration of arm function in order to maximize the effect of future approaches. Despite the increasing use of robotic systems in clinical and research settings, it is still questioned which of the wide variety of available robotic outcome measures are relevant to study arm movement ability following stroke.

### Outcome measures

Currently, therapy effectiveness is generally assessed with clinical scales. However, some clinical scales show a lack of reproducibility, in addition to subjectivity when scoring the test. One way to obtain objective and specific information concerning arm function at the body function level is to measure kinematics of the arm, as can be done by many upper extremity robotic systems. Recently, relations between active range of motion (aROM) and clinical scales as the Fugl-Meyer (FM) scale, the Chedoke McMaster Stroke Assessment score and the Stroke Impact Scale were studied [16]. Strong correlations were found between the FM scale and an aROM task, performed in the horizontal plane with the upper arm elevated to 90 degrees. A movement task highly similar to the aROM task used in [16] is circle drawing.

### Circle task

Successful circle drawing requires coordination of both the shoulder and elbow joint which makes it a potentially useful movement task to study multi-joint coordination. Dipietro et al. [17] showed that the effect of a robotic training intervention could be quantified by several outcome measures obtained during circular hand movements that were performed at table height. Because of the multi-joint nature of the movement task, circle drawing is a suitable task to study body functions [18] such as ranges of joint motion and coupling between the shoulder and elbow joint. In addition, circle area gives a quantitative description of the size of the region where someone can place his/her hand to grasp and manipulate objects. Such an outcome measure at the activity level gives functional information, in this case regarding the work space of the arm.

### Objective

The aim of this study is to examine whether circle drawing metrics are suitable outcome measures for objective assessment of upper extremity function of stroke survivors. A new method to objectively quantify the occurrence of synergistic movement patterns is introduced. Outcome measures will be compared between healthy subjects and stroke survivors to study the discriminative power between these groups. Within stroke survivors, correlations between outcome measures including the FM are addressed to study mutual dependencies.

## Methods

### Subjects

Chronic stroke survivors were recruited at rehabilitation centre 'Het Roessingh' in Enschede, the Netherlands. Inclusion criteria were a right-sided hemiparesis because of a single unilateral stroke in the left hemisphere and the ability to move the shoulder and elbow joints partly against gravity. Healthy elderly (45-80 years) were recruited at the research department and from the local community. Exclusion criteria for both groups were shoulder pain and the inability to understand the instructions given. All subjects provided written informed consent. The study was approved by the local medical ethics committee.

### Procedures

During a measurement session, subjects were seated on a chair with the arm fastened to an instrumented exoskeleton called Dampace [19]. This exoskeleton was only used for measurements and did not support the arm. Stroke subjects were asked to draw 5 and healthy subjects were asked to draw 15 consecutive circles during a continuous movement in both the clockwise (CW) and counter clockwise (CCW) direction. Circle drawing started with the hand close to the body, just above a tabletop of 75 cm height. The upper arm was aligned with the trunk and the angle between the upper arm and forearm was approximately 90 degrees. Templates of circles of different radii were shown on the tabletop to motivate subjects to draw the circles as big and as round as possible. To minimize the effect of compensatory trunk movements on the shape and size of the circles, the trunk of each subject was strapped with a four point safety belt. Movements were performed at a self selected speed, without touching the table. The order of direction of the circle drawing task (CW or CCW) was randomized across subjects.

### Measurements

Kinematic data were recorded with sensors integrated in the robotic exoskeleton [19]. Potentiometers on three rotational axes allowed measurements of upper arm elevation, transversal rotation, and axial rotation. A rotational optical encoder was used to measure elbow flexion and extension. Shoulder translations were measured with linear optical encoders. Signals from the potentiometers were converted from analog to digital (AD) by a 16 bits AD-converter (PCI 6034, National Instruments, Austin, Texas). The optical quadrature encoders were sampled by a 32 bits counter card (PCI6602, National Instruments, Austin, Texas). Digital values were sampled with a rate of 1 kHz, online low-pass filtered with a first order Butterworth filter with a cut-off frequency of 40 Hz and stored on a computer with a sample frequency of at least 20 Hz.

**Figure 1:**
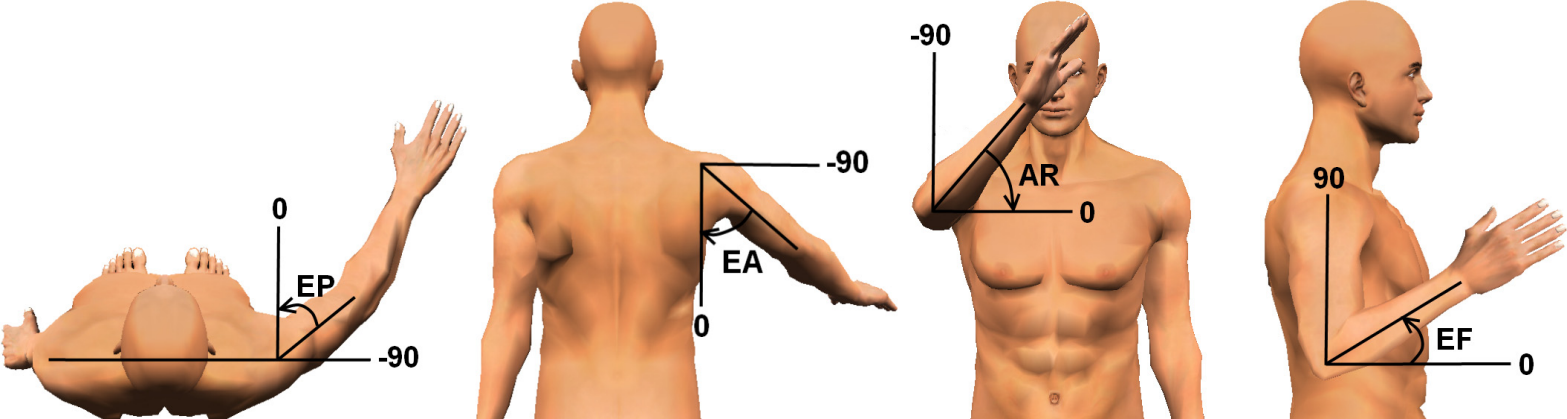
**Visual representation of the joint angles of the upper arm. Arrows indicate positive rotations**. EP = Elevation Plane, EA = Elevation Angle, AR = Axial rotation, EF = Elbow Flexion.

Arm segment lengths were measured to translate measured joint angles into joint positions. Upper arm length was measured between the acromion and the lateral epicondyle of the humerus. The length of the forearm was defined as the distance between the lateral epicondyle of the humerus and the third metacarpophalangeal joint. Thoracohumeral joint angles were measured according to the recommendations of the International Society of Biomechanics [20]. The orientation of the upper arm was represented by three angles, see Figure 1. The plane of elevation (EP) was defined as the angle between the humerus and a virtual line through the shoulders. The elevation angle (EA) represented the angle between the thorax and the humerus, in the plane of elevation. Axial rotation (AR) was expressed as the rotation around a virtual line from the glenohumeral joint to the elbow joint. The elbow flexion angle (EF) was defined as the angle between the forearm and the humerus. Joint excursions were calculated as the range between minimal and maximal joint angles during circle drawing.

Level of impairment of the hemiparetic arm of stroke survivors at the time of the experiment was assessed with the upper extremity part (max 66 points) of the FM scale [21]. Because the focus of the present study is on proximal arm function, a subset of the upper extremity part of the FM scale consisting of items A_II_, A_III _and A_IV _(max 30 points) was addressed separately (FMp).

### Data analysis

All measured signals were off-line filtered with a first order zero phase shift low-pass Butterworth filter with a cut-off frequency of 5 Hz. Joint positions were calculated by means of the measured shoulder displacement and successive multiplication of the measured joint angles and the transformation matrices defined for each arm segment. Joint positions were expressed relative to the shoulder position to minimize the contribution of trunk movements to the size and shape of the drawn circles.

Individual circles were extracted from the data between two minima of the Euclidean distance in the horizontal plane between the hand path and the shoulder position, which was represented in the origin. After visual inspection of the data for correctness and completeness, the three largest circles in both the CW and CCW direction were averaged and used for further analysis.

### Circle drawing metrics

**Figure 2:**
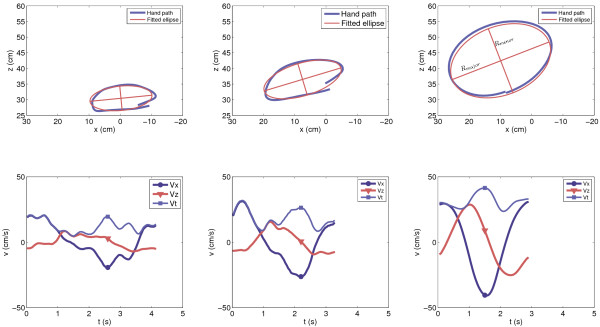
**Typical examples of hand paths (top) and corresponding speed profiles (bottom)**. Data from stroke survivors with FM = 9 (left), FM = 45 (middle) and a healthy subject (right). FM = Fugl-Meyer, Vx = speed in x-direction, Vz = speed in z-direction, Vt = tangential speed, Rmajor = major axis fitted ellipse, Rminor = minor axis fitted ellipse.

**Figure 3:**
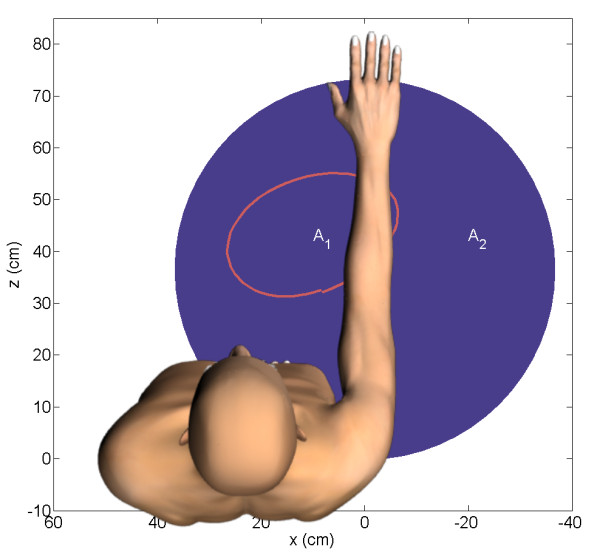
**Graphical representation of the calculation of the normalized work area (normA)**. The area (A_1_) enclosed by the hand path is divided by the area (A_2_) of a circle with a diameter equal to the length of the arm, measured between the acromion and the third metacarpophalangeal joint.

The area of the enclosed hand path reflects the active range of motion of both healthy subjects and stroke survivors, see Figure 2 for typical examples. Normalized circle area (normA) is expressed as ratio between the area of the enclosed hand path and the maximal circle area that is biomechanically possible to compensate for the effect of arm length on maximal circle area, see Figure 3. Circle area is considered maximal when the diameter of the circle equals the arm length of the subject.

Circle morphology was evaluated by calculation of the roundness as described in Oliveira et al. [22] and previously used to evaluate training induced changes in synergistic movement patterns during circle drawing of stroke survivors [23,17]. In this method, roundness is calculated as the quotient of the minor and major axes (see Figure 2) of the ellipse which is fitted onto the hand path by means of a principal component analysis. The calculated roundness lies between 0 and 1 and a perfectly round circle yields a roundness of 1.

To explicitly study the potential impact of synergistic movement patterns on circle drawing, movements within and out of the flexion and extension synergies were identified based on simultaneous changes in shoulder abduction/adduction (EA) and elbow flexion/extension (EF) angles. When the angular velocity of both shoulder abduction and elbow flexion exceeded 2% of their maximal values, movement was regarded as movement within the flexion synergy (InFlex). Movement within the extension synergy (InExt) was characterized by concurrent shoulder adduction and elbow extension, both exceeding the threshold value of 2% of the maximal angular velocity. In a similar way movement out of the flexion synergy (OutFlex) was characterized by simultaneous shoulder abduction and elbow extension, while movement out of the extension synergy (OutExt) comprised shoulder adduction and elbow flexion. If the angular velocity of one joint was below the threshold this was regarded as a single-joint movement (SJMov). InFlex and InExt represented movement within a synergistic pattern (InSyn). The ability to move out of a synergistic pattern (OutSyn) was calculated as the sum of OutFlex and OutExt.

### Statistical analysis

For statistical analysis, all data were tested for normality with the Kolmogorov-Smirnov test. Initial analysis revealed a small but statistically significant difference in age between both groups, see Table 1. For that reason, all outcome measures were tested for their ability to discriminate between healthy subjects and stroke survivors by means of analysis of covariance (ANCOVA) with fixed factor 'group' and covariate 'age'. Within-subject relations between outcome measures were identified and tested with Pearson's correlation coefficients. Correlations were considered weak when ρ < 0.30, moderate when 0.30 ≤ ρ ≤ 50 and strong when ρ > 0.50 [24]. The significance level for all statistical tests was defined as α = 0.05.

## Results

### Subjects

A total of 36 subjects, 20 healthy subjects and 16 stroke survivors, participated in this study. Characteristics of the subjects are summarized in Table 1. All stroke survivors had right-sided hemiparesis, which affected the dominant arm in all but one subject. All healthy subjects performed movements with the dominant arm. Stroke survivors were on average 4.8 years older than healthy subjects, p = 0.032. The effect of age on all outcome measures did not differ significantly between stroke survivors and healthy elderly, as indicated by non-significant interaction terms (group*age), p > 0.12.

**Table 1:** Subject demographic and clinical characteristics

	Healthy	Stroke
n	20	16
Age (yrs)	53.9 ± 5.3	58.7 ± 7.4
Gender	10 M/10 F	8 M/8 F
Dominance	20 R/0 L	15 R/1 L
Time post stroke (yrs)	-	3.3 ± 2.6
Fugl-Meyer (max 66)	-	33.4 ± 17.6 (7 - 60)
Fugl-Meyer proximal (max 30)	-	15.8 ± 8.5 (1 - 29)

Abbreviations:

M = male, F = female, R = right side, L = left side

### Circle metrics

Outcome measures were normally distributed in both healthy subjects (p ≥ 0.337) and stroke survivors (p ≥ 0.365) as indicated by the Kolmogorov-Smirnov test for normality. Group mean normA in healthy subjects was 34.6 ± 6.7%, which is significantly (p < 0.001) larger than the mean normA in stroke survivors, which was 12.8 ± 12.3% (see Figure 2 for typical examples). On average, roundness was significantly higher (p < 0.001) in the healthy group (0.66 ± 0.07) compared to the stroke survivor group (0.39 ± 0.17). Healthy subjects had significantly (p < 0.001) higher self selected movement speeds compared to stroke survivors (respectively 45.5 ± 8.6 and 16.2 ± 8.0 cm/s) and significantly (p < 0.001) shorter movement times to draw one circle (respectively 3.2 ± 0.9 and 7.8 ± 5.1 s).

### Joint excursions

**Figure 4:**
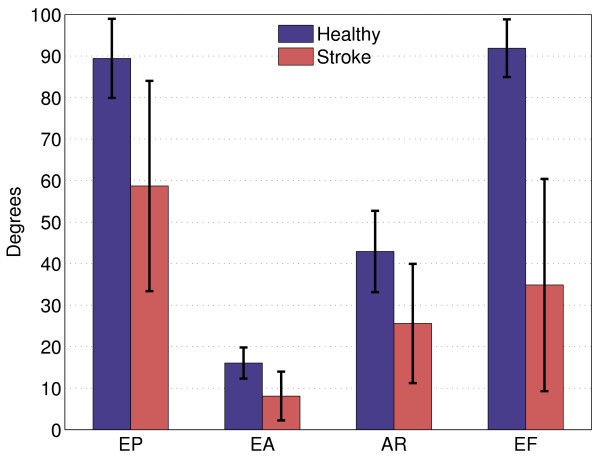
**Group mean joint excursions during circle drawing of healthy subjects and stroke survivors**. Error bars indicate one standard deviation. EP = Elevation Plane, EA = Elevation Angle, AR = Axial Rotation, EF = Elbow Flexion.

All measured joint excursions during circle drawing were significantly smaller (p < 0.001) in stroke survivors compared to the healthy subjects, see Figure 4. Healthy subjects varied EP on average 89.4 ± 9.5 degrees, against 58.7 ± 25.3 degrees for stroke survivors. The mean excursion of EA in healthy subjects was 16.1 ± 3.8 degrees, and 8.1 ± 5.9 degrees in stroke survivors. Mean variations in AR for healthy subjects and stroke survivors were respectively 42.9 ± 9.8 and 25.6 ± 14.3 degrees. EF was on average 91.9 ± 6.9 degrees in healthy subjects and 34.9 ± 25.5 degrees in stroke survivors.

### Synergistic movement patterns

**Figure 5:**
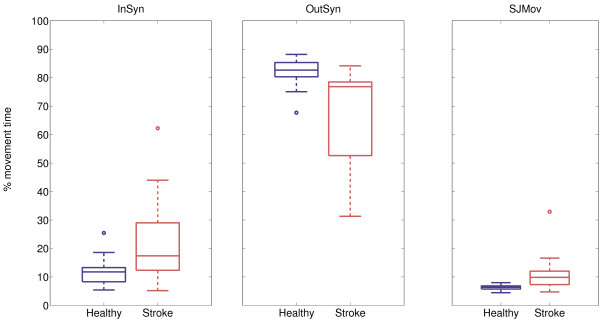
**Occurrence of synergistic movement patterns during circle drawing**. Boxplots of movement within (InSyn) or out of (OutSyn) synergistic movement patterns and single-joint movements (SJMov) of healthy subjects and stroke survivors.

The occurrence of synergistic movement patterns during circle drawing in both healthy subjects and stroke survivors are graphically displayed in Figure 5. Healthy subjects moved on average 11.5 ± 4.6% of the movement time within synergistic patterns, which was significantly (p = 0.005) less than stroke survivors, who moved during 22.2 ± 15.6% of the movement time within synergistic patterns. In the healthy group, OutSyn was on average 82.2 ± 4.7 percent which was significantly (p < 0.001) higher than in the stroke survivor group with mean OutSyn of 66.7 ± 16.6%. Finally, SJMov was on average 6.3 ± 0.9% in healthy subjects, and 11.1 ± 6.6% in stroke survivors, which is a statistically significant difference, p = 0.011.

### Relations between outcome measures

Pearson's correlation coefficients between the used outcome measures of stroke survivors are displayed in Table 2. The outcome measures used to describe the size and shape of the drawn circles are strongly related to the proximal part of the upper extremity portion of the FM scale (ρ = 0.86 and ρ = 0.79, respectively). Strong positive correlations can also be seen between the joint excursions and the size and shape of the circle (ρ ≥ 0.76).

**Table 2:** Pearson's correlation coefficients between outcome measures.

	FM	FMp	normA	rness	InSyn	OutSyn	SJMov	EP	EA	AR	EF
FM	1	0.97	0.79	0.75	-0.72	0.84	-0.41	0.63	0.58	0.63	0.83
FMp	0.97	1	0.86	0.79	-0.76	0.84	-0.33	0.77	0.68	0.72	0.90
normA	0.79	0.86	1	0.78	-0.56	0.62	-0.24	0.87	0.90	0.84	0.95
rness	0.75	0.79	0.78	1	-0.65	0.78	-0.44	0.76	0.79	0.87	0.91
InSyn	-0.72	-0.76	-0.56	-0.65	1	-0.92	-0.06	-0.61	-0.48	-0.49	-0.64
OutSyn	0.84	0.84	0.62	0.78	-0.92	1	-0.35	0.57	0.52	0.59	0.73
SJMov	-0.41	-0.33	-0.24	-0.44	-0.06	-0.35	1	0.01	-0.18	-0.33	-0.31
EP	0.63	0.77	0.87	0.76	-0.61	0.57	0.01	1	0.81	0.90	0.86
EA	0.58	0.68	0.90	0.79	-0.48	0.52	-0.18	0.81	1	0.85	0.87
AR	0.63	0.72	0.84	0.87	-0.49	0.59	-0.33	0.90	0.85	1	0.87
EF	0.83	0.90	0.95	0.91	-0.64	0.73	-0.31	0.86	0.87	0.87	1

Abbreviations:

FM = Fugl-Meyer, FMp = proximal part of upper extremity part of Fugl-Meyer, normA = normalized circle area, rness = roundness, InSyn = movement within synergistic pattern, OutSyn = movement out of synergistic pattern, SJMov = Single-Joint Movement, EP = Elevation Plane, EA = Elevation Angle, AR = Axial Rotation, EF = Elbow Flexion.

**Figure 6:**
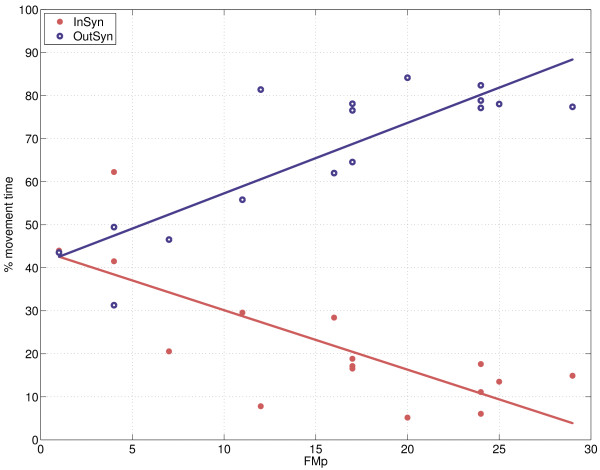
**Relation between the proximal part of the upper extremity part of the FM scale (FMp) and the occurrence of synergistic movement patterns**. InSyn = movement within synergistic pattern, OutSyn = movement out of synergistic pattern.

Movement within synergistic patterns is negatively correlated with FMp (ρ = -0.76), FM (ρ = -0.72), and the size and shape of the circles, ρ < -0.56, see Table 2 and Figure 6. InSyn is also negatively correlated with joint excursions (ρ < -0.48), indicating that subjects generally have smaller joint excursions when movement takes place within synergistic patterns. The ability to move out of synergistic movement patterns as indicated by OutSyn is positively correlated with the FMp (ρ = 0.84), FM (ρ = 0.84) and the size and shape of the circles (ρ > 0.62). Movement out of synergistic patterns is also positively correlated with joint excursions (ρ > 0.52).

## Discussion

In this study a standardized motor task and corresponding metrics were examined for discriminative power between healthy subjects and stroke survivors. Significant differences in normalized circle area, circle roundness, and the occurrence of synergistic movement patterns between healthy and stroke survivors were found, indicating the ability of these outcome measures to discriminate between these two groups. Also strong within-subject relations were found between several outcome measures in a sample of mildly to severely affected chronic stroke survivors.

### Work area

Reduced aROM during various movement tasks is commonly observed in stroke survivors, for example during planar pointing movements [25]. The present study indicates that joint excursions of the hemiparetic shoulder and elbow are diminished, resulting in a reduced work area of the hand. This finding is supported by studies of Sukal and Ellis [16,26] who showed a reduced work area of the paretic arm compared to the unaffected arm, during an aROM task with the upper arm elevated to 90 degrees (comparable to EA = -90 degrees in the present study).

### Roundness

Roundness of circles drawn by stroke survivors was previously studied by Dipietro and colleagues [23,17]. The method of determining roundness of a circle [22] was equal in the present study and the studies by Dipietro et al. During baseline measurements Dipietro et al. [17] found a mean roundness of 0.51 in a sample of 117 chronic stroke survivors with a mean FM score of 20.5. Mean roundness of the circles drawn by the chronic stroke survivors (mean FM 33.4 points) in the present study was 0.39, indicating that circles were more elliptical (i.e. less round). This was unexpected since a positive correlation coefficient (ρ = 0.76) between the FM score and roundness was found. A possible explanation for this discrepancy was already hypothesized in Dipietro et al., they measured subjects while the arm was supported against gravity. Application of gravity compensation reduces the activation level of shoulder abductors needed to hold the arm against gravity, and as a result the amount of coupled involuntary elbow flexion is decreased, leading to an increased ability to extend the elbow [6,27]. In the case of circle drawing, increase in aROM due to gravity compensation can lead to smaller differences in lengths of the major and minor axes of the fitted ellipse, resulting in higher values for roundness.

### Work area and FM

In the present study, a strong correlation between aROM, as represented by the normalized circle area, and the FM scale was found. Similar results were found in a study performed by Ellis et al [16]. In that study, aROM of stroke survivors during different limb loadings was measured. Movement was performed in the horizontal plane, with the upper arm elevated to 90 degrees. Correlation between aROM and FM varied with limb loading, and was 0.69 in the unsupported condition. In the present study, correlation between FM and normalized circle area was higher with a correlation coefficient of 0.79. The difference in correlation coefficients can be caused by differences in the performed movement task. During the study by Ellis et al. subjects were asked to make a movement as big as possible without instructions concerning the shape of the movement. Participants of the present study were asked to make circular movements as big and as round as possible. Also some differences in applied normalization procedures to minimize the effect of arm length on work area may contribute to differences in correlation between FM and aROM. Nevertheless, both studies showed strong relations between FM and aROM, indicating that circle area is a suitable outcome measure to objectively study activities of the upper extremity following stroke.

### Roundness and FM

Compared to the present study, Dipietro et al. [17] found similar, but less pronounced correlations between roundness and the FM scale (ρ = 0.55 against ρ = 0.75) and between roundness and the proximal upper extremity part of the FM scale (ρ = 0.61 against ρ = 0.79) during baseline and evaluation measurements. Because subjects in the study of Dipietro et al. drew circles in a gravity compensated environment, joint coupling during circle drawing is likely to be less pronounced compared to the unsupported arm movements that were made during the FM assessment, resulting in a less strong correlation between the FM score and circle roundness.

### Joint coupling and FM

Again, concerning the correlation between the FM and joint coupling, a comparison between Dipietro et al. [17] and the present study reveals a stronger correlation in the latter one, which is likely related to the use of gravity compensation in Dipietro et al.

Also, Dipietro et al. studied joint coupling by comparison of shoulder horizontal ab-/adduction (i.e. plane of elevation in the present study) and elbow flexion/extension angles whereas in the present study simultaneous changes in elevation angle and elbow angle represented joint coupling. A lower correlation between the proximal part of the FM scale and joint coupling as calculated by Dipietro et al. could also indicate that coupling between plane of elevation and elbow angle is less strong than coupling between elevation angle and elbow angle. This is supported by a smaller amount of secondary torque of elbow flexion measured during an isometric maximal voluntary contraction (MVC) of shoulder flexion (i.e. shoulder horizontal adduction) compared to an MVC of shoulder abduction [28]. Despite small differences in motor task, methods and analyses, both studies indicate that circle drawing is a suitable movement task to study coupling between two joints.

### Multi-joint movement

Compared to a rather strong focus on single-joint movements of the FM assessment, outcome measures concerning multi-joint movements are more suitable to study motor control during movements that resemble ADL tasks. Circle drawing is a multi-joint movement task that requires selective and coordinated movement of both the shoulder and elbow joint. At the activity level, normalized circle area gives a quantitative description of the size of the area where the stroke survivor can place his hand to grasp and manipulate objects. In addition, the measured joint excursions, the calculated roundness, and the occurrence of synergistic movement patterns quantify arm movement at the body function level. Drawing tasks are often used to study motor control of the arm during multi-joint movements, for example to study control of interaction torques between the shoulder and elbow joints [29,30].

As demonstrated in the present study and several other studies, circle size and roundness are strongly related to the widely used FM scale. This suggests that measurement of circle size and shape can give similar information about the level of impairment of stroke survivors. However, circle metrics are measured objectively and are insusceptible to subjective judgment by the examiner.

### Objective outcome measures

Quantitative outcome measures strongly related to pathological impairments can help to create a better understanding of neurological changes induced by post stroke rehabilitation therapy. Knowledge of size and shape of circular movements after stroke is extended in the present study by measurement of circle metrics in healthy subjects. The ability to compare changes of circle metrics induced by post stroke interventions with values obtained from a healthy population can provide insight in whether neural recovery takes place or whether stroke survivors use compensatory strategies. The degree to which both processes occur may influence future post stroke rehabilitation programmes [31].

A better understanding of mechanisms involved in post stroke rehabilitation is needed to maximize the effect of future approaches to improve upper extremity functionality. The use of standardized quantitative outcome measures allows a uniform comparison of different interventions to study their efficacy and identify which interventions are the most beneficial for stroke survivors.

### Clinical implications

Measurement of the use of synergistic patterns as described in this paper requires an advanced measurement system that is capable of measuring joint angles. These outcome measures can be useful to study underlying mechanisms of restoration of arm function after stroke in a research setting. Circle size and roundness can be measured not only with advanced measurement systems, but with any measurement device that is capable of measuring hand position. Besides advanced robotic systems, one can think of simple and affordable hand tracking devices, for instance based on a camera. Such equipment is suitable to deploy in clinical practice which allows simple but objective measurement of meaningful measures of arm function.

## Conclusions

The aim of this study was to examine whether circle drawing metrics are suitable outcome measures for stroke rehabilitation. The present study indicates that it is possible to make a distinction in circle area, roundness and the use of synergistic movement patterns between healthy subjects and stroke survivors with a wide range of stroke severity. These circle metrics are also strongly correlated to stroke severity, as indicated by the proximal upper extremity part of the FM score.

Outcome measures such as circle area and roundness can be a valuable addition to currently used outcome measures, because they can be measured objectively with any measurement device that is capable of measuring hand position. Such simple and affordable equipment is suitable to be deployed in clinical settings.

Identification of abnormal synergistic movement patterns requires more advanced equipment that is capable of measuring joint angles of the shoulder and elbow. Research into changes in the use of abnormal movement patterns is useful for a better understanding of mechanisms that are involved in restoration of post stroke arm function. Data obtained from healthy elderly can help to interpret changes in circle drawing metrics of stroke survivors, for instance to study effectiveness of post stroke interventions aiming at restoration of arm function.

## Competing interests

The authors declare that they have no competing interests.

## Authors' contributions

TK performed the design of the study, acquisition and analysis of data and drafting of the manuscript. BIM made substantial contributions to acquisition of the data and drafting of the manuscript. AH, JSR and JHB were involved in interpretation of results and critical revision of the manuscript for important intellectual content. JHB was also involved in conception and design of the study. GBP was involved in design of the study, acquisition and interpretation of data, drafting of the manuscript and critical revision of the manuscript for important intellectual content. All authors have read and approved the final manuscript.
